# Prediction of the origin of French Legionella pneumophila strains using a mixed-genome microarray

**DOI:** 10.1186/1471-2164-14-435

**Published:** 2013-07-01

**Authors:** Jeroen W Den Boer, Sjoerd M Euser, Nico J Nagelkerke, Frank Schuren, Sophie Jarraud, Jerome Etienne

**Affiliations:** 1 Regional Public Health Laboratory Kennemerland, Boerhaavelaan 26, 2035 RC, Haarlem, the Netherlands; 2 Department of Community Medicine, United Arab Emirates University, Al Ain, United Arab Emirates; 3 Department of Microbiology, Netherlands Organisation for Applied Scientific Research TNO, Zeist, Netherlands; 4 Centre National de Référence des Légionelles, Lyon, France

**Keywords:** Legionnaires’ Disease, Legionella Pneumophila, Virulence-associated Epitope, Pneumonia, Negative Predictive Value, Random Forest Algorithm, Environmental Exposure, Genomotyping, Micro-array, Public Health

## Abstract

**Background:**

*Legionella* is a water and soil bacterium that can infect humans, causing a pneumonia known as Legionnaires’ disease. The pneumonia is almost exclusively caused by the species *L*. *pneumophila*, of which serogroup 1 is responsible for 90% of patients. Within serogroup 1, large differences in prevalence in clinical isolates have been described. A recent study, using a Dutch *Legionella* strain collection, identified five virulence associated markers. In our study, we verify whether these five Dutch markers can predict the patient or environmental origin of a French *Legionella* strain collection. In addition, we identify new potential virulence markers and verify whether these can predict better. A total of 219 French patient isolates and environmental strains were compared using a mixed-genome micro-array. The micro-array data were analysed to identify predictive markers, using a Random Forest algorithm combined with a logistic regression model. The sequences of the identified markers were compared with eleven known *Legionella* genomes, using BlastN and BlastX; the functionality for each of the predictive markers was checked in the literature.

**Results:**

The five Dutch markers insufficiently predicted the patient or environmental origin of the French *Legionella* strains. Subsequent analyses identified four predictive markers for the French collection that were used for the logistic regression model. This model showed a negative predictive value of 91%. Three of the French markers differed from the Dutch markers, one showed considerable overlap and was found in one of the *Legionella* genomes (Lorraine strain). This marker encodes for a structural toxin protein RtxA, described for *L*. *pneumophila* as a factor involved in virulence and entry in both human cells and amoebae.

**Conclusions:**

The combination of a mixed-genome micro-array and statistical analysis using a Random Forest algorithm has identified virulence markers in a consistent way. The Lorraine strain and related Dutch and French *Legionella* strains contain a marker that encodes a RtxA protein which probably is involved in the increased prevalence in clinical isolates. The current set of predictive markers is insufficient to justify its use as a reliable test in the public health field in France. Our results suggest that genetic differences in *Legionella* strains exist between geographically distinct entities. It may be necessary to develop region-specific mixed-genome microarrays that are constantly adapted and updated.

## Background

Legionnaires’ disease (LD) is an acute pneumonia of low incidence, comprising 3-6% of community-acquired pneumonia, depending on the study population [1,2]. The causative bacterium belongs to the genus *Legionella*, an accidental human pathogen that can replicate in alveolar macrophages, although it normally replicates as an intracellular parasite in protozoa. *Legionella* needs an aquatic environment to survive and is therefore cultured from natural or man made waters as well as from wet soil and sewage sludge. Not all environmental *Legionella* bacteria seem to have the potential to infect humans. From all known species and serogroups, strains that are typed as *L*. *pneumophila* serogroup 1 are the most prevalent in clinical isolates. Several studies [3,4] suggested a further division into strains that do or do not react to monoclonal antibody MAb3/1, with the former group supposedly more virulent. In these studies, virulence was explored by comparing causative strains derived from immuno-competent and immuno-compromised LD patients. Heterogeneity in virulence has also be studied by comparing the genotype distributions of environmental strains with those of clinical *Legionella* isolates. Several studies have found large differences between these distributions, suggesting that different virulence factors exist [5,6]. For example, in the Netherlands 50% of the LD patients is caused by three genotypes that represent 4% of the patient-related environmental distribution [7]. Using genomotyping to identify virulence markers, a recent study described four markers that could discriminate clinical isolates and environmental strains in a systematic collection of 222 Dutch *Legionella* strains [8].

In the present paper, we report on the genomotyping of a systematic collection of 219 French strains

using the same techniques. We evaluate the performance of a logistic regression model based on the four Dutch markers to adequately predict the origin of 114 clinical *Legionella* isolates and 105 environmental strains from France. Furthermore, based on the genetic data from the French strains, using the Random Forest algorithm and logistic regression, we identify predictive markers for French clinical versus French environmental origin and compare these to the four Dutch markers that were identified earlier.

## Results and discussion

The prediction model with the four Dutch markers, when applied to the French strains, had a sensitivity (for being of clinical origin), specificity, NPV and PPV prediction of 78%, 54%, 70% and 65%, respectively (see Table 1). As this indicates that the predictive value of the Dutch makers is much lower for the French strains, an optimized novel marker set for the French strains was selected.

**Table 1:** **Prediction results on the origin of 219 French *Legionella *strains using five Dutch markers**

**Complete dataset**	**Origin of strains**		**Total**
	**Clinical**	**Environmental**	
Prediction clinical	89	48	137
Prediction environmental	25	57	82
Total	114	105	219
Sensitivity	78%		
Specificity	54%		
PPV	65%		
NPV	70%		

*Abbreviation*: *PPV* positive predictive value, *NPV* negative predictive value.

The development of a new prediction model, based on the genetic data from the French strain collection resulted in a model that consisted of four genetic markers, three of which differ from the four Dutch markers. In order to maximize the sensitivity of the model (predict as many of the clinical strains correctly), while minimizing the loss of specificity, we used an arbitrary probability (of classifying a strain as clinical) cut-off value of 0.2 to classify the alternative outcomes. The 109 strains in the eleventh “test dataset” (that were not present in the eleventh training dataset that was used to develop the model) were predicted by the model with a sensitivity of 96%, a specificity of 38%, a PPV of 63%, and a NPV of 91% (see Table 2).

**Table 2:** Prediction results for the 109 strains in the eleventh “test dataset” using four French markers

**Test dataset**	**Origin of strains**		**Total**
	**Clinical**	**Environmental**	
Prediction clinical	55	32	87
Prediction environmental	2	20	22
Total	57	52	109
Sensitivity	96%		
Specificity	38%		
PPV	63%		
NPV	91%		

*Abbreviation*: *PPV*, positive predictive value ; *NPV*, negative predictive value.

Three of the four French markers that were selected differed from the Dutch markers, although some of them were strongly correlated (see Table 3). This indicates that although there is a limited overlap (one marker) between the selected Dutch and French predictive markers, some markers are strongly related to each other (which could explain their presence in either one of the final prediction models) (Additional file 1).

**Table 3:** Spearman correlation coefficients of the 4 Dutch and the 4 French predictive markers for the total sample of French strains (n = 219)

	**Dutch markers**				**French markers**			
	**7B8**	**15D6**	**16E4**	**33 F8**	**8D12**	**12H3**	**19D6**	**27B10**
7B8	1	0.421**	0.212**	0.209**	−0.183**	−0.381**	0.416**	−0.060
15D6	0.421**	1	0.177**	0.462**	−0.110	−0.432**	0.859**	−0.199**
16E4	0.212**	0.177**	1	0.082	0.073	−0.085	0.228**	0.116
33 F8	0.209**	0.462**	0.082	1	−0.327**	−0.048	0.492**	0.060
8D12	−0.183**	−0.110	0.073	−0.327**	1	0.195**	−0.080	0.153*
12H3	−0.381**	−0.432**	−0.085	−0.048	0.195**	1	−0.439**	0.230**
19D6	0.416**	0.859**	0.228**	0.492**	−0.080	−0.439**	1	−0.169*
27B10	−0.060	−0.199**	0.116	0.060	0.153*	0.230**	−0.169*	1

Correlation coefficients are based on the hybridization ratios of the individual markers.

* p-value < 0.05, **p-value < 0.01.

French marker 19D6 has substantial overlap with Dutch marker 15D6 (sequence locations are 713550–714251 versus 713281–713837, respectively (Table 4)) [8]. The difference in marker hybridization ratio between clinical isolates and environmental strains is shown in Table 5. The effect sizes (the difference in ratio divided by the within group standard deviation of the ratio in the total sample) for markers 33 F8 (Dutch model) and 27B10 (French model) were relatively small compared to the other 6 markers, suggesting a limited influence of these markers in the calculated prediction of the final models (Table 5). The sequence analyses of the four selected markers showed that all markers were present in one or more of the completely sequenced *Legionella* isolates (Table 4).

**Table 4:** **Sequence location of the four predictive markers in available *Legionella *genomes**

**Legionella strains**	**Predictive markers**			
	**8D12**	**12H3**	**19D6**	**27B10**
Paris	2915168-2914552	2003186-2002613	n/a	674697-673883
Philadelphia	2812796-2812180	2027502-2026929	n/a	607471-606657
Lens	2748941-2748325	1985753-1985180	n/a	658953-658139
Corby	2990951-2990335	2108162-2107589	n/a	698878-698064
Alcoy	2927471-2926855	2102218-2101645	n/a	689774-688960
130b	2885344-2884728	1975530-1974957	n/a	683508-682754
Lorraine	2804908-2805524	1928210-1928783	713550-714251	628527-629341
HL 060401035	2910715-2911331	2131955-2132528	n/a	656754-657568
L. longbeachae NSW150	n/a	n/a	n/a	n/a
L. longbeachae D-4968	n/a	n/a	n/a	n/a
ATCC 43290	2773461-2772845	1943894-1943321	n/a	611624-610810

n/a = not applicable.

**Table 5:** Table of differences in hybridization ratios for the 4 Dutch and the 4 French markers between clinical isolates and environmental strains

**Markers**	**Hybridization ratios**			
	**Total sample**	**Clinical isolates**	**Environmental strains**	**Effect size**
	**(n = 219)**	**(n = 114)**	**(n = 105)**	
Dutch markers				
7B8	1.64 (2.94)	2.33 (3.26)	0.89 (2.35)	0.50
15D6	0.50 (0.53)	0.64 (0.53)	0.34 (0.48)	0.60
16E4	1.17 (0.25)	1.23 (0.16)	1.11 (0.30)	0.50
33 F8	1.08 (0.36)	1.07 (0.39)	1.09 (0.34)	−0.06
French markers				
8D12	0.96 (0.17)	1.01 (0.13)	0.91 (0.19)	0.57
12H3	0.89 (0.20)	0.84 (0.16)	0.94 (0.23)	−0.50
19D6	0.64 (0.83)	0.91 (0.87)	0.35 (0.68)	0.72
27B10	1.05 (0.14)	1.04 (0.09)	1.05 (0.18)	−0.11

Data represent mean hybridization ratios (SD). Effect size was calculated as the difference in ratio between clinical isolates and environmental strains, divided by the within group standard deviation (SD) of the ratio in the total sample. SD = standard deviation.

Marker 19D6 is encoding for a structural toxin protein RtxA. This function is proposed to play a role in both adherence and entry into *Acanthamoeba castellanii* similar to that observed in human monocytic cells. Furthermore, it was found that RtxA is involved in intracellular survival and trafficking [9,10]. When comparing the nucleotide sequences of French marker 19D6 and Dutch marker 15D6 with the NCBI nucleotide database by megablast for highly similar sequences, the only hit is a region in the *L*. *pneumophila* Lorraine genome (Genbank ID FQ958210.1) described as the RtxA toxin. In the region, ranging from nucleotides 711260–716600, 11 hits are obtained, clearly indicating the repeat character of this sequence which has also been described by Gomez-Valero et al. [11]. Changing the blast settings for more dissimilar sequences and somewhat similar sequences does not result in additional hits, indicating the unique presence of this sequence in a specific subset of *L*. *pneumophila* strains. Comparing sequences with the NCBI nucleotide database by BlastX, in which the provided sequence is translated in all reading frames which are then compared to the translated nucleotide database, additional information is obtained. The only high homology hits again are related to the *L*. *pneumophila* Lorraine genome (Genbank ID FQ958210.1), but also low homology hits can be observed. These include bacterial genera such as *Methylobacterium*, *Pseudomonas* and *Rhizobium* and at an even lower homology level also other *Legionella* strains such as the Philadelphia and Paris strains.

Marker 8D12 is associated with homospermidine synthase: This function is mainly encountered in bacteria which are involved in interactions with eukaryotic organisms, especially in plant pathogens and symbionts. Homologues of this sequence are mainly encountered in alpha proteobacteria and apart from *L*. *pneumophila*, *Pseudomonas syringae*/*aeruginosa* are the only gamma proteobacteria containing this function. Horizontal gene transfer to alpha proteobacteria is proposed [12].

The sequence of marker 12H3 is predicted to encode an ATP dependent DNA helicase/uvr/REP helicase, an enzyme that makes single stranded DNA from double stranded DNA. This function is better represented within gamma proteobacteria than 8D12.

Marker 27B10 is encoding for tyrosyl tRNA synthetase. This function is commonly present in gamma proteobacteria and its standard function is related to tRNA production. In *Enterococcus* species this gene however is also part of the tyramine biosynthesis cluster (a naturally occurring monoamine compound) so other functions for this gene cannot be excluded [13].

We have demonstrated that the predictive markers derived from a Dutch strain collection insufficiently predict the strain origin in a similar collection from France. We thus developed a “French” prediction model for predicting the origin (clinical or environmental) of French *L*.*pneumophila* strains, based on four different markers. The NPV of this model (91%) seems insufficient to justify its use as a reliable test in the public health field in France, for this purpose the NPV should be higher. NPV’s should be well above 95%, similar to recently published screening tests for methicillin-resistant *Staphylococcus aureus* (MRSA) and extended-spectrum-beta- lactamase-producing Enterobacteriaceae (ESBL) [14,15].

It is interesting that the predictive markers for the Netherlands and France differ as only one of the four Dutch markers showed substantial overlap with one of the four French markers, although some of them showed significant correlations. This finding suggests genetic differences in *Legionella* strains between geographically distinct entities. This is in line with earlier studies showing differences between Northern and Southern European countries in the occurrence of *L*. *pneumophila* serogroup 1 strains that are positive for the virulence associated monoclonal antibody MAb 3/1 from the so called Dresden panel [4].

The overlapping markers encode for a structural toxin protein RtxA, that has been described for *L*. *pneumophila* as a factor involved in virulence and entry in both human cells and amoebae [9,10]. Furthermore, more detailed analyses of RtxA proteins in *L*. *pneumophila* have been published elsewhere [16-18]. Although there appear to be highly homologous regions in the sequences of this protein our data clearly show that the Lorraine strain and related Dutch and French strains contain a significantly different RtxA protein which probably is involved in the increased virulence of these strains.

There may be several reasons why our approach for identifying predictive markers for clinical isolates was more successful in The Netherlands than in France. One of them is that our original shotgun library was exclusively derived from Dutch strains, thus insufficiently reflecting the genetic diversity in France. To avoid such bias, it may well prove necessary to develop region specific mixed-genome microarrays.

Perhaps also, genotypic differences between clinical *Legionella* isolates and environmental strains could differ between The Netherlands and France. Differences that may well have been introduced by selection biases, as the Dutch environmental strains were collected within the National *Legionella* Outbreak Detection Programme (NLODP) and only derived from patient-related sources during source investigations, while the French environmental strains, although partly collected similarly, also included strains that were collected differently, and are not directly related to LD patients.

Another reason could be that, in view of the modest sample sizes included, the combination of genomotyping with the subsequent statistical method is not sufficiently reproducible or robust as stochastic factors play a major role in the identification of strains used for classification.

## Conclusions

The combination of a mixed-genome micro-array and statistical analysis using a Random Forest algorithm has identified virulence markers in a consistent way. We identified four genetic markers that can be used to predict the patient or environmental origin of French *L*. *pneumophila* strains. At least one of the markers, encoding for structural toxin protein RtxA, had previously already been identified as being associated with virulence. Our data clearly show that the Lorraine strain and related Dutch and French strains contain marker that encodes for a significantly different RtxA protein which probably is involved in the increased prevalence in clinical isolates. The NPV of a potential test using these four markers is high, but inadequate for use as a public health screening tool in France. Our results suggest that genetic differences in *Legionella* strains exist between geographically distinct entities. It may be necessary to develop region-specific mixed-genome microarrays that are constantly adapted and updated.

## Methods

### Strain collection

Since 1998, all clinical strains isolated in France are sent to the National Reference Centre for *Legionella* (NRC-L) for characterization and molecular analysis. For this study, a sample of 114 *L*. *pneumophila* serogroup 1 clinical strains isolated from hospitalized patients with community-acquired LD in metropolitan France was selected during the period 2006–2009. Clinical isolates from nosocomial LD patients and from LD patients who had traveled ≥ 5 days abroad during their incubation period, were excluded.

Environmental isolates are also sent to NRC-L for identification during routine environmental control or for typing by using Sequence-Based Typing method, PFGE method and monoclonal antibody sub-grouping, allowing the identification of source of contamination. For this study, a random sample of 105 environmental *L*. *pneumophila* strains isolated from the period 2003–2008 was selected, including 91 *L*. *pneumophila* sg 1 and 14 *L*. *pneumophila* non-serogroup 1 isolates. These strains were collected throughout France from hot water system, cooling towers, sludge, fountains and thermal waters. The origin of the environmental strains allowed classifying the isolates in 5 groups: (a) related to an outbreak (5 strains); (b) found in a source related to a LD patient and matched with the patient isolate (on SBT and PFGE level) (1 strain); (c) found in a source related to a LD patient, but not matched with the patient isolate (20 strains); (d) not related to LD patients (76 strains); (e) endemic strains sharing a previously observed genotype and responsible for at least 30 epidemiologically unrelated cases of legionellosis (3 strains).

### Microarray development

The mixed-genome microarray used in this study has been described elsewhere, and was developed using a systematic collection of Dutch *Legionella* strains [19]. In short, four clinical *Legionella* isolates and four environmental *L*. *pneumophila* strains were selected based on their diversity to provide a shotgun library. The library, consisting of 3360 genomic fragments was used to compare the labeled genomic DNA of 257 *Legionella* strains in the Dutch collection. The data for all spots were calculated as ratios between the tester strain and the library strains. Since 80% of the 3360 markers were present in all strains, encompassing the core genome, these were ignored for analysis. The remaining 20% showed considerable variation among strains. Also, where multiple markers showed nearly identical patterns over the complete data set (suggesting partial overlap or close linkage in the genome), only one was used for further analysis. As a result, 480 potentially relevant markers were used to develop the prediction model.

For the French collection the Dutch library was used to compare the labelled genomic DNA of 219 French strains. (Additional file 2).

### Prediction of the origin of French *Legionella* strains using Dutch markers

The method to identify genetic markers that predict the clinical or environmental origin of *Legionella* strains from a Dutch strain collection has been described elsewhere [8]. The method is based on a classification algorithm that uses an ensemble of different classification trees, called Random Forest. The Random Forest statistical approach performs excellent in infectious disease data classification problems [20,21]. In short, the Random Forest algorithm was applied on ten random training datasets to select 25 markers with the highest rank of “importance” in the prediction of the origin of the strains (clinical or environmental). From 250 markers (25 markers times 10 training datasets) the 25 most frequent genetic markers were chosen. An eleventh so called “training dataset” was constructed consisting of a random selection of clinical isolates and environmental *Legionella* strains. Using forward logistic regression, a model was developed for the “training dataset” with the 25 genetic markers entered as independent variables, and the origin of the strains (clinical or environmental) as dependent variable. The model was tested with a so-called ‘test dataset’ consisting of those strains that were not present in the eleventh training dataset, in order to prevent overfitting of the model. As a result, four Dutch markers were identified, that could predict the clinical or environmental origin of *Legionella* strains.

In our study, we examined whether the four Dutch markers, using the same logistic regression model, could predict the clinical or environmental origin of the French strains.

### Marker selection for *L*. *pneumophila* strains from France

Additionally, we developed a new prediction model for the French strain collection, using the same “Dutch” methodology [8]. The 219 French *L*. *pneumophila* strains were randomly assigned to 10 different training datasets, each consisting of 57 clinical and 53 environmental strains. For all training datasets, the 25 best predicting genetic markers were selected with the aid of the Random Forest algorithm. From these 250 markers, the 25 most common markers were chosen and entered in the logistic regression model that was developed for the eleventh constructed training dataset. This model was tested with the eleventh so-called ‘test dataset’, consisting of the 57 clinical and 52 environmental strains that were not used for the construction of the prediction model. The performance of the prediction model is presented in 2×2 tables, together with the estimated sensitivity, specificity, negative predictive value (NPV), and positive predictive value (PPV). The NPV is the most relevant test characteristic in public health situations in which our prediction would be useful [14,15].

### Comparison of the Dutch and French markers

The association between the four Dutch markers that were previously identified [8], and the predictive markers that were selected for the new prediction model based on the French strain collection was investigated by calculating the correlation between the hybridization ratios of the 4 Dutch and the 4 French predictive markers, using Spearman correlation coefficients. Additionally, the hybridization ratios of both the Dutch and the French markers were compared between clinical isolates and environmental strains. This was done by calculating the difference in hybridization ratio between clinical isolates and environmental strains, divided by the within group standard deviation (SD) of the ratio in the total sample. All analyses were performed using PASW Statistics 18.0, SPSS inc., Chicago, Illinois).

### Functionality of markers

The sequences of the markers that were selected in the final prediction model that was developed for the French strain collection were compared with the known *Legionella* sequences of eleven completely sequenced strains present in the NCBI database (http://www.ncbi.nlm.nih.gov): *Legionella longbeachae* (strains NSW150 and D-4968), *L. pneumophila* strains Paris, Philadelphia, Lens, Corby, Alcoy, 130b, Lorraine, HL 0604 1035, and ATCC 43290 using BlastN and BlastX.

## Competing interests

The authors declare that they have no competing interests.

## Authors’ contributions

JB, SE, NN, FS, SJ and JE participated in the design of the study. JB, SE and NN, participated in the statistical analysis. FS developed the microarray, collected the microarray data, and performed data processing. SJ and JE collected the bacterial strains. JB drafted the manuscript. All authors read and approved the final manuscript.

## Supplementary Material

Additional file 1 **Sequences of the four markers that best predict the patient or environmental origin of French *Legionella pneumophila *strains.** Click here for file

Additional file 2 **Hybridization ratios for 480 markers of the 219 French *Legionella pneumophila *strains.** Click here for file
